# Supplementary material for: Image-based force inference by biomechanical simulation
Source: PLoS Comput Biol. 2024 Dec 2;20(12):e1012629. doi: 10.1371/journal.pcbi.1012629 (PMC11637313; doi:10.1371/journal.pcbi.1012629)
Supplement: S1 Appendix — (PDF) [file pcbi.1012629.s001.pdf]

Supplementary Information

Supplementary text

Text A: DCM force balance

Here, we provide the details of the deformable cell model, based on Cuvelier et al. [1]. Cells behave as pressurized liquid "bubbles" with surface tension, adhesive tension, and internal pressure. The actomyosin cortex of a cell is represented by a viscous shell which is approximated by a triangulated surface mesh with effective thickness t_c, and nodal positions $\mathbf{x}_i$. In the following sections we will discuss different force contributions and their implementation.

Surface tension

Tension generated in the cortex results from actomyosin contractility, which is represented as an effective surface tension γ in the shell model. Adhesive tension ω_{ij} for contacting cells i, j explains the reduction of contractility in interfacial surfaces, as well as the interfacial adhesion energy. Hence, the net surface tension τ of cell i is given by

$$\tau_i = \begin{cases} \gamma_i & \text{for free surfaces} \\ \gamma_i - \omega_i & \text{for cell-cell contacting surfaces.} \end{cases}$$

The interfacial tension γ_{ij} is the sum of the two contacting individual cortex tensions as

$$\gamma_{ij} = \gamma_i + \gamma_j - \omega_i - \omega_j = \gamma_i + \gamma_j - \omega_{ij}$$

where adhesive tension per cell is chosen such that the force at the cell-cell junction is also balanced at the level of the individual cell in the interface plane

$$\omega_i = \gamma_i \left(\cos(\theta_{iI}) + 1\right)$$
$$\omega_j = \gamma_j \left(\cos(\theta_{jI}) + 1\right).$$

We compute $\cos(\theta_{iI})$ and $\cos(\theta_{jI})$ from the balance of forces at the cell-cell junction

$$\gamma_i \cos(\theta_{iI}) + \gamma_j \cos(\theta_{jI}) + \gamma_{ij} = 0$$
$$\gamma_i \sin(\theta_{iI}) - \gamma_j \sin(\theta_{jI}) = 0.$$

We get

$$\cos(\theta_{iI}) = \frac{-\gamma_i^2 + \gamma_j^2 - \gamma_{ij}^2}{2\gamma_i\gamma_{ij}}$$
$$\cos(\theta_{jI}) = \frac{\gamma_i^2 - \gamma_j^2 - \gamma_{ij}^2}{2\gamma_j\gamma_{ij}}$$
$$\cos(\theta_{ij}) = \frac{-\gamma_i^2 - \gamma_j^2 + \gamma_{ij}^2}{2\gamma_i\gamma_j}.$$

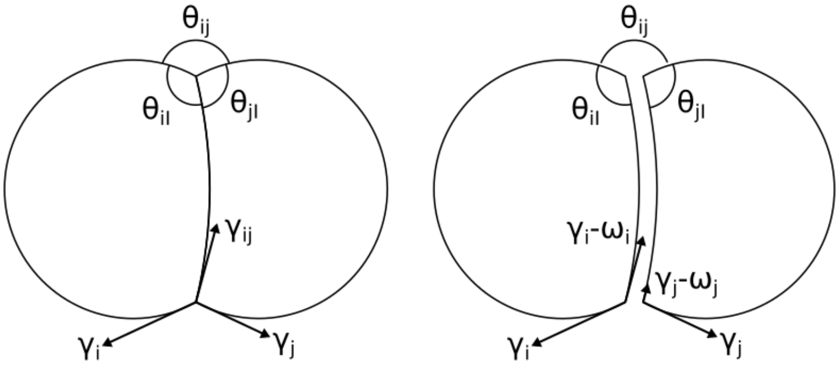

Figure A: Interfacial and adhesive tension at the cell-cell interface. Where two cells connect, we consider adhesive tension ω_{ij} equivalent to interfacial tension γ_{ij} when surface tensions γ_i, γ_j at free surfaces are known.

Therefore, the free parameters in the system become γ_i, γ_j, and γ_{ij} (or total adhesive tension $\omega_{ij} = \omega_i + \omega_j$ that we report in the manuscript). We have previously shown that our model predicts correct curvature and junction contact angles for varying adhesive tensions and surface tensions in Cuvelier et al. [1].

Using the derivation of Fedosov et al. [2], the direct force contribution of surface tension τ of node i is given by

$$\mathbf{F}_i^{\text{act}} = \frac{\tau}{2}(\mathbf{x}_k - \mathbf{x}_j) \times \hat{\mathbf{n}}_A, \tag{1}$$

where i, j, k are nodes of a single triangle $A = (ijk)$.

Internal pressure

Since the cytoplasm is assumed to be incompressible, active volume control is implemented via a cytoplasmic pressure. We assume the equilibrium volume V_α^* of a cell α is constant and implemented a proportional-integral (PI) pressure controller to enforce this. The cytoplasmic pressure $P_\alpha^{\text{cyt}}(t)$ due to the volume controller is then estimated as

$$P_\alpha^{\text{cyt}}(t) = -K\epsilon_\alpha(t) - \frac{K}{T_I}\int_0^t \mathrm{d}s\ \epsilon_\alpha(s), \tag{2}$$

for a cell with volumetric strain $\epsilon_\alpha(t) = (V_\alpha(t) - V_\alpha^*)/V_\alpha^*$. T_I is typically chosen as one order of magnitude larger than the time-resolution with which the system is updated. The total pressure acting on a node

$$P_i(t) = \frac{2\gamma}{R_\alpha^{\text{cell}}} + P_\alpha^{\text{cyt}}(t),$$

includes an additional offset pressure $2\gamma/R_\alpha^{\text{cell}}$ to balance the actomyosin contractility in the cortex. This ensures cells are in mechanical equilibrium at the start of the simulations. The force acting on node i due to nodal pressure $P_i(t)$, is obtained by integrating over

the node associated oriented Voronoi area S_i. Given the pressure is constant over the cell surface, the force is simply given by

$$\mathbf{F}_i^{\text{cyt}}(t) = \mathbf{S}_i \, P_i(t). \tag{3}$$

Medium damping

A general drag force

$$\mathbf{F}_i^{\text{drag}} = -\Gamma_{ii}^{\text{f}} \cdot \mathbf{v}_i,$$

where

$$\Gamma_{ii}^{\text{f}} = \frac{3\eta^{\text{f}}}{2R^{\text{cell}}} S_i \mathbb{I}, \tag{4}$$

is included to account for the liquid drag between the cells and their medium due to the fluid viscosity η^{f}. When dealing with arbitrary shapes this approximation is no longer correct. Even though F_i^{drag} is small compared to other dissipative forces, it is still used to improve the stability of the numerical integration scheme as it ensures the resistance matrix is positive definite, see further in equation of motion. [3]

Cortex viscosity

A much larger contribution to energy dissipation arises from cortex viscosity η. The viscous damping force between two connected nodes is given by

$$\mathbf{F}_{ij}^{\text{visc}} = \Gamma_{ij}^{c}(\mathbf{v}_j - \mathbf{v}_i), \tag{5}$$

with friction elements

$$\Gamma_{ij}^{\text{c}} = \frac{t_c \eta}{\sqrt{3}} \mathbf{I},$$

and $\mathbf{I}$ identity.

Wet contact friction

A viscous contact force is included to account for drag between contacting surfaces with friction constant ξ (units of Pa·s/m), expressing the scaling between a dissipative traction $\mathbf{T}_c$ and the sliding velocity of two contacting surfaces A and B

$$\mathbf{T}_{AB}^{\text{fric}} = -\xi \Delta \mathbf{v}_{AB}. \tag{6}$$

In the particle-based model, the contact drag force acting on node i of triangle A of the contact pair (AB) is computed as

$$\mathbf{F}_{AB,i}^{\text{fric}} = \Gamma_{AB}^{\text{fric}} \cdot \sum_{k \in B} w_{ik}^{AB} \, (\mathbf{v}_k - \mathbf{v}_i) \tag{7}$$

determined by a friction tensor $\Gamma_{AB}^{\text{fric}}$ and weights w_{ik}^{AB} per node k of the B triangle. w_{ik}^{AB} are assumed to scale with the relative contribution of the nodal contact forces to the overall contact force thus

$$w_{ik}^{AB} = \frac{\left(\mathbf{F}_{AB,i}^{\text{adh}} + \mathbf{F}_{AB,k}^{\text{adh}}\right) \cdot \hat{\mathbf{n}}_{AB}}{6 \sum\limits_{\forall k \in B} \mathbf{F}_{AB,k}^{\text{adh}} \cdot \hat{\mathbf{n}}_{AB}}$$

is used as an approximation. $\Gamma_{AB}^{\text{fric}}$ for a given contact area S_{AB} is estimated as

$$\Gamma_{AB}^{\text{fric}} = S_{AB}\left[\xi^{\perp}\hat{\mathbf{n}}_{AB}\otimes\hat{\mathbf{n}}_{AB} + \xi^{\parallel}\left(\mathbb{I} - \hat{\mathbf{n}}_{AB}\otimes\hat{\mathbf{n}}_{AB}\right)\right],$$

with normal and tangential friction coefficients $\xi^{\perp}$ and $\xi^{\parallel}$ respectively. Note that (7) can be equivalently formulated as

$$\mathbf{F}_{AB,i}^{\text{fric}} = \sum_{k\in B}\Gamma_{AB,ik}^{\text{fric}}\cdot\left(\mathbf{v}_k - \mathbf{v}_i\right),$$

if we denote

$$\Gamma_{AB,ik}^{\text{fric}} = w_{ik}^{AB}\Gamma_{AB}^{\text{fric}}. \tag{8}$$

Contact forces

In the mechanical cell model, we assume a fixed and uniform adhesive tension ω across the cell surface. The adhesive tension incorporates cell-cell adhesive interactions as well as the effect of cortical tension reduction at the cell-cell interface [4, 5, 6]. The total adhesive tension ω acting on interface (AB) is given by $\omega = \omega_A + \omega_B$. A linear force, scaling with contact overlap δ, was introduced to represent repulsive interactions between surfaces and ensure stable contacts. The contact potential for contact-pair (AB) with contact area S_{AB} is then given by

$$E_{AB}^{\text{adh}} = \left(\frac{\delta_{AB}}{h_0} - \frac{\delta_{AB}^2}{2h_0^2}\right)\omega S_{AB}$$

where a distinction can be made between the attractive and repulsive energy contributions respectively. The effective range of adhesion h_0 was incorporated by virtually translating contact surfaces along their normal direction. The contact overlap distance δ_{AB} is calculated with respect to these translated surfaces. The resulting contact pressure P_{AB}^{adh} is estimated with a linear contact pressure model where no energy is stored in deformation at equilibrium

$$P_{AB}^{\text{adh}}\left(\mathbf{x}\right) = k_{AB}\delta_{AB}\left(\mathbf{x}\right) - P_{AB}^{0},$$

contact stiffness k_{AB} and adhesive pressure P_{AB}^{0} need to be set. To ensure that at equilibrium ($P_{AB}^{\text{adh}}\equiv 0$) the work of adhesion ω is recovered, contact stiffness has to be defined as $k_{AB} = P_{AB}^{0}/h_0$ with $P_{AB}^{0} = \omega/h_0$. The specific value h_0 does not influence simulation outcome due to the scale separation, $h_0 \ll R^{\text{cell}}$. This is valid for all performed simulations as the effective range of adhesion h_0 was estimated as 300 nm. [7] Contact overlap distance at point $\mathbf{x}$ was defined as

$$\delta_{AB}\left(\mathbf{x}\right) = \max\left\{0;\ 2\left(\mathbf{x} - \mathbf{s}_{AB}\right)\cdot\left(\hat{\mathbf{n}}_{AB}\times\hat{\mathbf{l}}_{AB}\right)\tan\theta\right\},$$

where θ is the contact angle, $\hat{\mathbf{n}}_{AB}\propto\left(\hat{\mathbf{n}}_B - \hat{\mathbf{n}}_A\right)\times\hat{\mathbf{l}}_{AB}$ the contact normal and $\mathbf{s}_{AB}$ is an arbitrary point on the intersection line defined by $\hat{\mathbf{l}}_{AB}\propto\hat{\mathbf{n}}_A\times\hat{\mathbf{n}}_B$, as reported by Smeets et al. [8] The resulting contact forces and moment are obtained by integrating the total contact pressure over the oriented contact area $\mathbf{S}_{AB}$,

$$\mathbf{F}_{AB}^{\text{adh}} = \int \mathrm{d}\mathbf{S}_{AB}(\mathbf{x})\ P_{AB}^{\text{adh}}(\mathbf{x}). \tag{9}$$

To distribute the contact forces acting on the contact plains to the nodal contact forces $\mathbf{F}_{AB,i}^{\text{adh}}$, it is assumed that the nodal forces are collinear with the contact normal $\hat{\mathbf{n}}_{AB}$. This results in a system of linear equations per contact pair (AB)

$$\sum_{i\in A} \mathbf{F}^{\text{adh}}_{AB,i} = - \int\limits_{\mathbf{x}\in A\cap B} \mathrm{d}\mathbf{S}_{AB}(\mathbf{x})\; P^{\text{adh}}_{AB}(\mathbf{x})$$

$$\sum_{i\in A} [(\mathbb{I} - \hat{\mathbf{n}}_{AB} \otimes \hat{\mathbf{n}}_{AB}) \cdot (\mathbf{x}_i - \mathbf{x}_{AB})] \times \mathbf{F}^{\text{adh}}_{AB,i} = \int\limits_{\mathbf{x}\in A\cap B} \mathrm{d}\mathbf{S}_{AB}(\mathbf{x}) \times (\mathbf{x} - \mathbf{x}_{AB})\; P^{\text{adh}}_{AB}(\mathbf{x}) \tag{10}$$

which guarantees a unique solution for every $\mathbf{F}^{\text{adh}}_{AB,i}$. The contact point $\mathbf{x}_{AB}$ is defined as the geometric center of the intersection polygon $A \cap B$. [8] The integrals on the right-hand side are calculated by using a numerical quadrature rule presented in [9] with 7 quadrature points covering each triangle of the triangulated intersection polygon $A \cap B$ obtained as an intersection of projected triangles on a common plane. The precise choice of the number of quadrature points has no impact on the results in the simulation, as long as adequate quadrature coordinates and weighting values are chosen. The solution for this system is presented and discussed in the work of Smeets et al. [8].

This same contact model is used for cell-eggshell contacts where we assume low adhesive tension (ω_h), and high contact stiffness ($k_{AB} = \frac{1}{3}10^5 \frac{2\gamma}{R_0^2}$). The eggshell itself is assumed undeformable, meaning we don't integrate the node positions.

Protrusion forces

The protrusion model is defined by two spherical coordinates directing $\mathbf{p}_p$, an angle α defining the protrusion width and a total protruding force F_p. We model a protrusive pressure in direction $\mathbf{p}_p$ per node i at the front end of the cell by the difference of Gaussian kernels as

$$P_i^P = \beta_P \left(2 \exp \frac{-\theta_i^2}{2\alpha^2} - \exp \frac{-\theta_i^2}{8\alpha^2} \right). \tag{11}$$

Here β_P is the scaling factor determining the total protrusive force F_P, α gives a 3D angle indicating the width of protrusion pressure (Figure B), and θ_i is the 3D angle for every node between the line from the cell center x_c in direction $\mathbf{p}_p$ and the line $\mathbf{x}_i - \mathbf{x_c}$ such that

$$\cos\theta_i = \frac{\mathbf{p}_p \cdot (\mathbf{x}_i - \mathbf{x}_c)}{||\mathbf{p}_p||\, ||(\mathbf{x}_i - \mathbf{x}_c)||}. \tag{12}$$

We set β_P such that the total pushing force in direction $\mathbf{p}_p$ becomes

$$F_p = \int P_i^P H(P_i^P) \mathbf{p}_p \cdot d\mathbf{S}_i, \tag{13}$$

with the Heaviside function

$$H(P_i^P) = \begin{cases} 1\,, & P_i^P >= 0 \\ 0\,, & P_i^P < 0 \end{cases} \tag{14}$$

To make sure that the net force applied from protrusion is zero, we add a reaction pressure

$$P_i^R = \beta_R \exp \frac{-\theta_i^2}{8\alpha^2} \tag{15}$$

and set the reaction pressure scale β_R such that

$$0 = \int \left(P_i^P + P_i^R \right) \mathbf{p}_p \cdot d\mathbf{S}_i. \tag{16}$$

The force from cell protrusion per node i is computed as

$$\mathbf{F}_i^P = \mathbf{S}_i P_i^P. \tag{17}$$

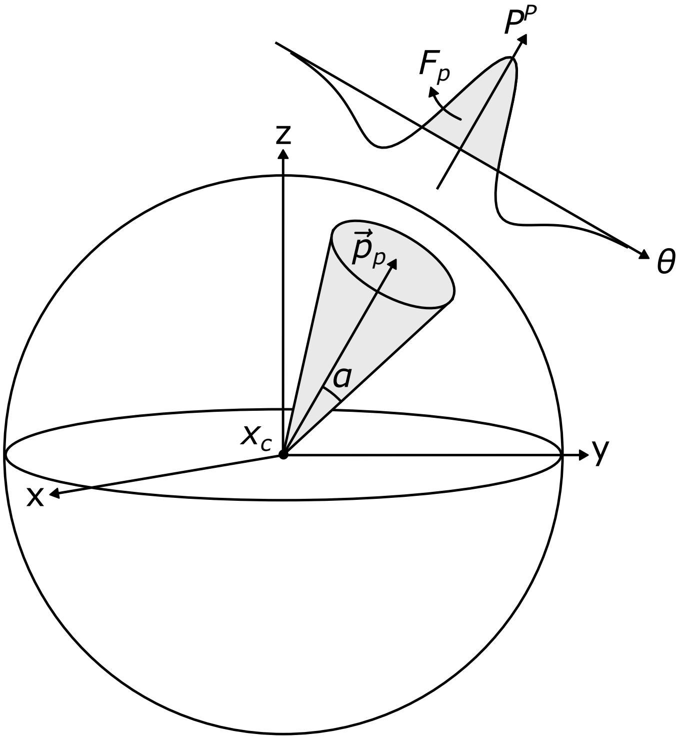

Figure B: Protrusion model. The protrusion is characterized by an orientation vector $\mathbf{p}_p$, angle α, and total protruding force F_p.

Cytokinetic ring forces

The cytokinetic ring model is defined by two spherical coordinates directing $\mathbf{p}_R$, spring constant k, and an offset distance d. The cell division plane is defined through point $\mathbf{x}_c + d\,\mathbf{p}_R$ with orientation $\mathbf{p}_R$, giving it an offset from cell center x_c. We add spring forces between the connecting nodes i, j of the contractile ring as

$$\mathbf{F}_i^R = k \left(1 - \frac{\Delta x_0}{\Delta x_R} \right) (\mathbf{x}_j - \mathbf{x}_i) \tag{18}$$

where k is the effective spring stiffness, Δx_0 is the constant reference length of the ring chosen as 20µm, and Δx_R is the instantaneous length of the contractile ring. Furthermore, since we observe in vivo that cytokinesis gives rise to a smooth contractile band, we add a smoothing force acting on ring nodes i and neighboring nodes j as

$$\mathbf{F}_j^{R,S} = \mathbf{p}_i \frac{k}{c} \left[\mathbf{p}_i \cdot (\mathbf{x}_i - \mathbf{x}_j) \right] \tag{19}$$

with c set to 5m. To make sure that we have a net-zero force balance

$$\mathbf{F}_i^{R,S} = -\mathbf{F}_j^{R,S} \tag{20}$$

with $\mathbf{p}_i = (\mathbf{x}_i - \mathbf{x}_c)/||\mathbf{x}_i - \mathbf{x}_c||$ the direction from the cell center to the ring node.

To keep the volumes of emerging daughter cells stable, we add pressure per daughter based on surface area deviation

$$P^D = K_D(S_D^* - S_D(t)) \tag{21}$$

with K_D a proportional controller constant ($1 \times 10^{12}\,\mathrm{Pa\,m^{-2}}$), S_D^* the equilibrium area and $S_D(t)$ the instantaneous area. The force per daughter node i becomes

$$\mathbf{F}_i^D = \mathbf{S}_i P_i^D. \tag{22}$$

Equation of motion

In the overdamped cellular environment, inertial forces may be neglected [10]. Based on the different contributions described above, the force balance for node i gives

$$\begin{aligned}\mathbf{F}_i^{\mathrm{act}} + \mathbf{F}_i^{\mathrm{cyt}} + \mathbf{F}_i^{P} + \mathbf{F}_i^{R} + \mathbf{F}_i^{R,S} + \mathbf{F}_i^{D} + \sum_{(AB):i\in A} \mathbf{F}_{AB,i}^{\mathrm{adh}} \\ = \Gamma_{ii}^{\mathrm{f}} \cdot \mathbf{v}_i + \sum_j \Gamma_{ij}^{\mathrm{c}} \cdot (\mathbf{v}_i - \mathbf{v}_j) + \sum_{(AB):i\in A} \sum_{k\in B} \Gamma_{AB,ik}^{\mathrm{fric}} \cdot (\mathbf{v}_i - \mathbf{v}_k)\end{aligned}$$

which can be abbreviated as

$$\mathbf{F}_i = \sum_j \Gamma_{ij} \cdot \mathbf{v}_j \tag{23}$$

for a system consisting of N nodes, where

$$\Gamma_{ij} = \begin{cases} \Gamma_{ii}^{\mathrm{f}} + \sum_{k:k\neq i} \Gamma_{ik}^{\mathrm{c}} + \sum_{(AB):i\in A} \sum_{k\in B} \Gamma_{AB,ik}^{\mathrm{fric}}, & i = j, \\ -\Gamma_{ij}^{\mathrm{c}} - \sum_{(AB):i\in A, j\in B} \Gamma_{AB,ij}^{\mathrm{fric}}, & i \neq j. \end{cases}$$

The Cartesian components of the overall force vectors can be represented as a single ($3N \times 1$) column matrix, while the friction matrices can be assembled to a single ($3N \times 3N$) sparse symmetric positive definite friction matrix [11]. The conjugate gradient method (CGM) is used to efficiently solve the system for the node velocities $\{\mathbf{v}_j\}$ at each iteration. Positions of the nodes are subsequently updated using a semi-implicit Euler integration scheme.

$$\mathbf{x}_i(t + \Delta t) = \mathbf{x}_i(t) + \Delta t\, \mathbf{v}_i(t + \Delta t), \tag{24}$$

where $\mathbf{v}_i(t + \Delta t)$ are the projected new velocities obtained by solving eq. 23 at time t via the CGM.

Text B: Protein signal correction

First, the raw protein signal from a microscopy time-lapse is smoothed using a Gaussian kernel with a sigma of 0.2 microns. The smoothed signal is then projected onto the cell meshes, where each mesh node takes on the value of the nearest pixel.

To account for bleaching over time and signal weakening in z, a linear generalized additive model (GAM) is applied. Time (t) and z are fitted to piecewise second-order splines using the following pyGAM syntax:

```
gam = LinearGAM(s('t', n_splines=10, spline_order=2)
              + s('z', n_splines=20, spline_order=2))
```

The signal is subsequently normalized to a range of 0 to 1.

For calculating the protein value at a cell-cell interface or for an entire cell, a simple average of the corresponding mesh nodes is used.

Text C: Automatic detection of protrusions and cytokinetic rings

Both rings and protrusions can be detected and assigned automatically, based on a quantitative analysis of the cell shape. To demonstrate this, we used FlowShape [12] to set up a pipeline for the detection of protrusions and cytokinetic rings, along with their orientation. We selected a representative set of 31 cell shapes, which were then manually labeled with the number of protrusions. Further, a cell was labeled as having a cytokinetic ring when it has divided in the next frame in the time-lapse.

The protrusion detector is a Laplacian of Gaussian filter to detect regions of high curvature, followed by a simple threshold (the same filter as described in van Bavel et al. [12]). This design reached an accuracy of 90% on our dataset. To detect cytokinetic rings, we made a synthetic 'template' of a dividing cell, and try to match it to all cells by maximizing the cross-correlation. Using a single template of a symmetric division, we managed to match all but one cell, giving an accuracy of 97%. The missed cell shows a highly asymmetric division, and could additionally be correctly classified by introducing a second template that accounts for asymmetry. The matched templates also contain information about the orientation of the division plane, but we did not quantify these. Code and data for this pipeline can be found in the FlowShape repository: `https://bitbucket.org/pgmsembryogenesis/flowshape/src/main/demo/detect_features.ipynb?viewer=nbviewer`.

Supplementary tables

Condition	Velocity	Decay	Experiments	Markers
P0-A	18.7	0.50	19	1498
P0-P	10.1	0.54	14	738
ABal	10.5	0.55	7	420
ABar	9.7	0.43	11	602
$ABpl_{DV}$	13.5	0.47	18	1137
$ABpl_{AP}$	7.2	0.64	7	296
ABpr	8.5	0.48	21	1098
E	13.9	0.71	21	1175
MS	8.0	0.49	21	1332
P2	13.7	0.87	14	794

Table A: Numeric ablation results and sample sizes.

Element	V	P	R	Deviation (°)	Total force (µN)	N
P0 ring	0.00	1.00	−0.02	0	0.008 ± 0.007	3
AB ring	−0.86	0.51	0.00	12	0.76 ± 0.14	7
P1 ring	−0.02	0.99	−0.12	4	0.29 ± 0.17	8
ABa ring	−0.53	0.07	0.85	5	0.46 ± 0.02	5
ABp ring	−0.38	0.21	0.9	10	0.27 ± 0.16	7
EMS ring	0.10	0.95	−0.28	3	0.26 ± 0.03	3
P2 ring	0.93	0.37	−0.01	11	0.36 ± 0.17	9
ABpl ventral protrusion	0.96	0.13	0.23	10	0.0034 ± 0.0025	24
ABpl anterior protrusion	−0.23	−0.97	0.07	10	0.0032 ± 0.0017	24
ABpl dorsal lamellipodium	−0.93	−0.36	0.09	14	0.0018 ± 0.0019	24
ABpr ventral protrusion	0.99	−0.10	−0.13	9	0.0032 ± 0.0021	24

Table B: Averaged parameters of local force generating activities. The 3D coordinate system is aligned to the embryo symmetry axes, with V representing the ventral side of the DV axis, P the posterior side of the AP axis, and R the right side of the LR axis. For cytokinetic rings, only data from the last 2 minutes before division is used. The orientation of a ring is a unit vector perpendicular to the division plane, while the total force on the ring is defined as spring constant k, multiplied by ring length. Local protrusions are averaged over the entire 7-cell stage. The total force is the pushing force on the protrusion, while the orientation is given as a unit vector pointing from the cell center to the protrusion tip. For all features, the orientation is a weighted average of the individual orientations, using the total force they exerted as weights. The variation of orientations is given as an angular weighted standard deviation, using the same weights.

Parameters	Symbol	Synthetic	PiCS	FIDES	Units	Sources
Surface tension	γ	0.75	1.5	0.75	$\mathrm{nN\,\mu m^{-1}}$	[13, 1]
Adhesion cell-cell	ω_{ij}	0.75	0.5	0.5	$\mathrm{nN\,\mu m^{-1}}$	[11, 13]
Adhesion cell-hull	ω_h	0.01	0.01	0.01	$\mathrm{nN\,\mu m^{-1}}$	assumed minimal
Bulk modulus cell	K	1.5	1	2	kPa	[1]
K integrative term	T_I	$2\Delta t$	$2\Delta t$	$2\Delta t$	s	
Thickness cortex	t_c	300	300	300	µm	[14, 1]
Adhesive range	h	200	250	200	nm	
Normal Friction	$\xi^{\perp}$	1	1	1	$\mathrm{kPa\,s\,\mu m^{-1}}$	
Tangential Friction	$\xi^{\parallel}$	0.05	0.01	0.1	$\mathrm{kPa\,s\,\mu m^{-1}}$	
Liquid viscosity	η^{f}	25	500	25	Pa s	[13]
Cortex viscosity	η	2	2	2.5	kPa s	
Cell radius	R_0	10	10	10	µm	
Simulation timestep	Δt	0.05	0.04	0.05	s	stable
Simulation duration		150	1.5	2.5	s	

Table C: Mpacts physical parameters. The synthetic parameters are used to generate synthetic embryos. PiCS parameters are used for the final relaxation step of PiCS image segmentation. FIDES parameters are used for the stability tests of input meshes.

References

[1] Maxim Cuvelier, Jef Vangheel, Wim Thiels, Herman Ramon, Rob Jelier, and Bart Smeets. Stability of asymmetric cell division: A deformable cell model of cytokinesis applied to c. elegans. *Biophysical Journal*, 122(10):1858–1867, May 2023. doi: 10.1016/j.bpj.2023.04.017. URL https://doi.org/10.1016/j.bpj.2023.04.017.

[2] Dmitry A. Fedosov, Bruce Caswell, and George Em Karniadakis. Systematic coarse-graining of spectrin-level red blood cell models. *Computer Methods in Applied Mechanics and Engineering*, 199(29–32):1937–1948, June 2010. ISSN 0045-7825. doi: 10.1016/j.cma.2010.02.001. URL http://dx.doi.org/10.1016/j.cma.2010.02.001.

[3] P. Van Liedekerke, B. Smeets, T. Odenthal, E. Tijskens, and H. Ramon. Solving microscopic flow problems using stokes equations in sph. *Computer Physics Communications*, 184(7):1686–1696, July 2013. ISSN 0010-4655. doi: 10.1016/j.cpc.2013.02.013. URL http://dx.doi.org/10.1016/j.cpc.2013.02.013.

[4] Jean-Léon Maître, Hélène Berthoumieux, Simon Frederik Gabriel Krens, Guillaume Salbreux, Frank Jülicher, Ewa Paluch, and Carl-Philipp Heisenberg. Adhesion functions in cell sorting by mechanically coupling the cortices of adhering cells. *Science*, 338(6104):253–256, October 2012. ISSN 1095-9203. doi: 10.1126/science.1225399. URL http://dx.doi.org/10.1126/science.1225399.

[5] Hervé Turlier and Jean-Léon Maître. Mechanics of tissue compaction. *Seminars in Cell & Developmental Biology*, 47–48:110–117, December 2015. ISSN 1084-9521. doi: 10.1016/j.semcdb.2015.08.001. URL http://dx.doi.org/10.1016/j.semcdb.2015.08.001.

[6] Carl-Philipp Heisenberg and Yohanns Bellaïche. Forces in tissue morphogenesis and patterning. *Cell*, 153(5):948–962, May 2013. ISSN 0092-8674. doi: 10.1016/j.cell.2013.05.008. URL http://dx.doi.org/10.1016/j.cell.2013.05.008.

[7] J. Israelachvili. *Intermolecular and Surface Forces, 3rd Edition*. 06 2011.

[8] Bart Smeets, Tim Odenthal, Simon Vanmaercke, and Herman Ramon. Polygon-based contact description for modeling arbitrary polyhedra in the discrete element method. *Computer Methods in Applied Mechanics and Engineering*, 290:277–289, June 2015. ISSN 0045-7825. doi: 10.1016/j.cma.2015.03.004. URL http://dx.doi.org/10.1016/j.cma.2015.03.004.

[9] Linbo Zhang, Tao Cui, and Hui Liu. A set of symmetric quadrature rules on triangles and tetrahedra. *Journal of Computational Mathematics*, 27:89–96, 01 2009.

[10] E. M. Purcell. Life at low reynolds number. *American Journal of Physics*, 45(1):3–11, January 1977. ISSN 1943-2909. doi: 10.1119/1.10903. URL http://dx.doi.org/10.1119/1.10903.

[11] Tim Odenthal, Bart Smeets, Paul Van Liedekerke, Engelbert Tijskens, Hans Van Oosterwyck, and Herman Ramon. Analysis of initial cell spreading using mechanistic contact formulations for a deformable cell model. *PLoS Computational Biology*, 9(10):e1003267, October 2013. doi: 10.1371/journal.pcbi.1003267. URL https://doi.org/10.1371/journal.pcbi.1003267.

[12] Casper van Bavel, Wim Thiels, and Rob Jelier. Cell shape characterization, alignment, and comparison using flowshape. *Bioinformatics*, 39(6), June 2023. ISSN

1367-4811. doi: 10.1093/bioinformatics/btad383. URL http://dx.doi.org/10.1093/bioinformatics/btad383.

[13] Maxim Cuvelier, Jiří Pešek, Ioannis Papantoniou, Herman Ramon, and Bart Smeets. Distribution and propagation of mechanical stress in simulated structurally heterogeneous tissue spheroids. *Soft Matter*, 17(27):6603–6615, 2021. doi: 10.1039/d0sm02033h. URL https://doi.org/10.1039/d0sm02033h.

[14] Bart Smeets, Maxim Cuvelier, Jiri Pešek, and Herman Ramon. The effect of cortical elasticity and active tension on cell adhesion mechanics. *Biophysical Journal*, 116(5): 930–937, March 2019. doi: 10.1016/j.bpj.2019.01.015. URL https://doi.org/10.1016/j.bpj.2019.01.015.
