# Supplementary figures and images for: Image-based force inference by biomechanical simulation
Source: PLoS Comput Biol. 2024 Dec 2;20(12):e1012629. doi: 10.1371/journal.pcbi.1012629 (PMC11637313; doi:10.1371/journal.pcbi.1012629)

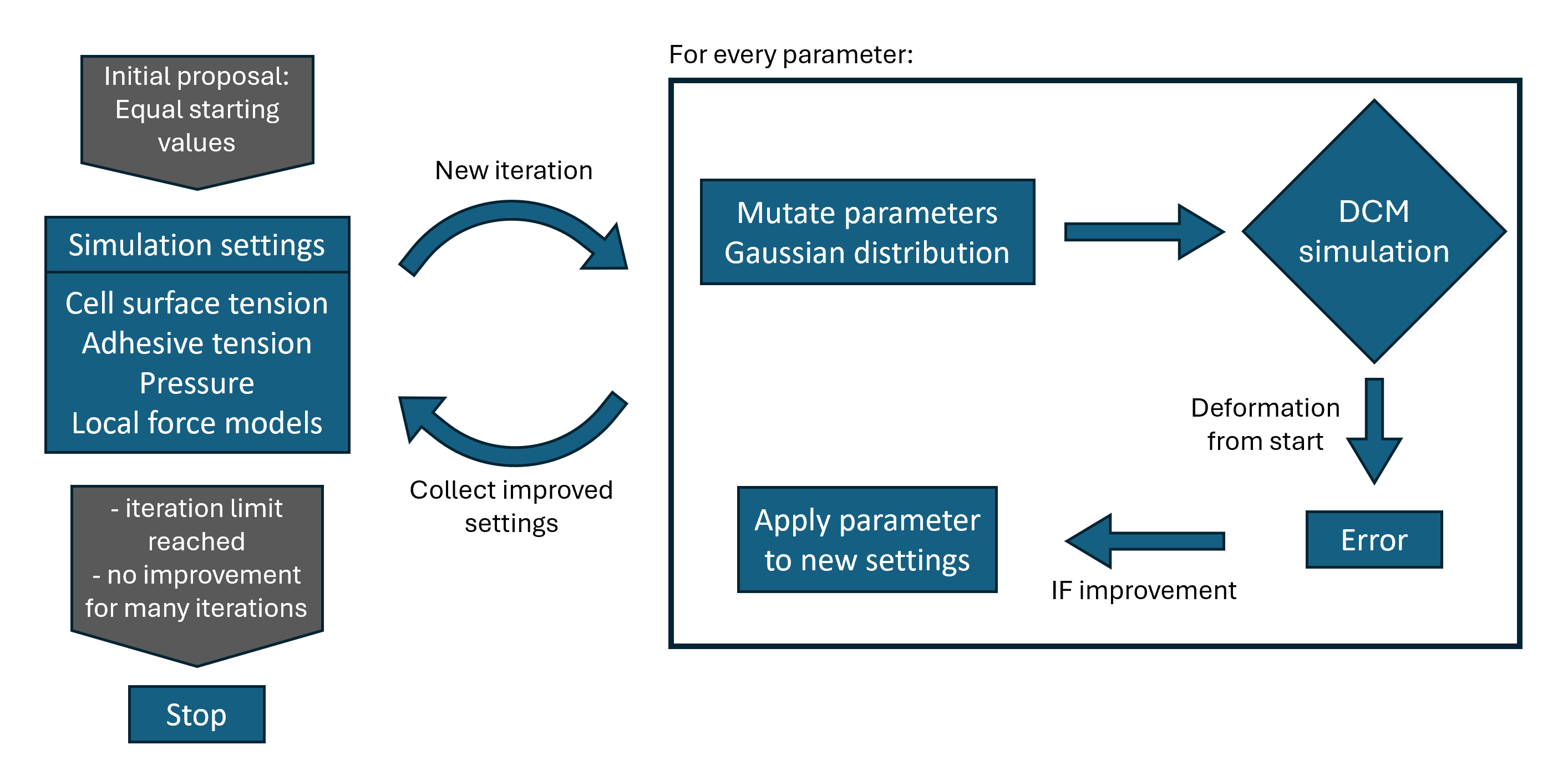

Supplement: S9 Fig — Repeated iterations evaluate parameter changes by computing the deformation error in a DCM simulation. Gaussian learning rate and bounds for all parameters can be found in the code (solve_shape.py). (TIFF) [file pcbi.1012629.s010.tiff]
